# HighSTEPS: A High Strain Temperature Pressure and Speed Apparatus to Study Earthquake Mechanics

**DOI:** 10.1007/s00603-021-02362-w

**Published:** 2021-02-04

**Authors:** M. Violay, C. Giorgetti, C. Cornelio, F. Aeschiman, G. Di Stefano, L. Gastaldo, S. Wiemer

**Affiliations:** 1 Laboratory of Experimental Rock Mechanics EPFL, Lausanne, Switzerland; 2 MEquadrat AG, Technopark Luzern, Platz 4, 6039 Root D4, Switzerland; 3 Istituto Nazionale Di Geofisica E Vulcanologia, Rome, Lazio Italy; 4 Department of Earth Sciences, ETH Zürich, Sonneggstrasse 5, 8092 Zurich, Switzerland

**Keywords:** Biaxial friction apparatus, Low to high slip velocity, Deformation conditions of the seismogenic upper crust

## Introduction

Physics of earthquake source can be investigated by monitoring active faults from borehole observatory in reservoirs (Maxwell et al. 2010) or by interpretation of seismic waves at the earth’s surface (Shearer 2019). Indeed, most information on earthquake mechanics is retrieved from seismology (e.g., Lee et al. 2002). However, the low resolution of these indirect techniques (cm to km scale) yields limited information on the physical and chemical deformation mechanisms active during earthquake rupture nucleation and propagation (Kanamori and Anderson 1975).

Experimental studies of frictional instabilities on fault gouge material or pre-existing surfaces (e.g., Brace and Byerlee 1966) may overcome those limitations (Scholz 1998; Marone 1998; Persson 2013). For instance, friction controls earthquake nucleation and propagation, the static and dynamic stress drops, the frictional heat generated during slip, and consequently the energy budget of earthquakes (Scholz 2019; Di Toro et al. 2011). All these processes can be investigated and monitored through laboratory experiments. In the last decades, rock friction properties have long been investigated using triaxial apparatuses in saw-cut configuration (e.g., Jaeger 1959; Byerlee 1967; Handin 1969), in which the fault is loaded at low velocities, typically orders of µm/s, and accumulates small displacements, typically few mm. In a seminal paper, Brace and Byerlee (1966) suggested that the stick–slip phenomenon observed in these rock friction experiments is analogous to natural earthquakes. Furthermore, to address the problem of earthquakes nucleation, biaxial apparatuses were developed and have long been used to study frictional properties of experimental faults under sub-seismic slip velocities in double-direct shear configuration (e.g., Dieterich 1972; Mair et al. 2002; Collettini et al. 2014; Giorgetti et al. 2015). The biaxial apparatus developed at USGS (USA) is amongst the first biaxial apparatuses used to investigate rock frictional properties (e.g., Dieterich 1972). Other pioneering biaxial apparatuses are the one in the Rock and Sediment Mechanics Laboratory at the Pennsylvania State University (USA) (e.g., Mair et al. 2002) and BRAVA (Brittle Rock deformAtion Versatile Apparatus) installed at INGV in Rome (Italy) (Collettini et al. 2014). Although the biaxial apparatuses developed in the past 50 years are characterized by different boundary conditions in terms of forces, pressures, temperatures and size of the samples, all of them take advantages from the double-direct shear configuration that allows good control of the normal and shear forces acting of the fault, accurate measurements of fault slip and dilation/compaction, and constant contact area.

Friction studies conducted with triaxial and biaxial deformation apparatuses are characterized by sub-seismic slip velocities and a limited amount of slip, < 10^–3^ m/s and few cm, respectively (e.g., Jaeger 1959; Byerlee 1967,1978; Brace and Byerlee 1966; Handin 1969; Paterson and Wong 2005; Lockner and Beeler 2002; Mair et al. 2002; Savage and Marone 2007; Samuelson et al. 2009; Carpenter et al. 2016). These experiments showed that the apparent static friction coefficient μ (i.e., *μ* = *τ*/*σ*_n_^eff^, where τ is the shear stress and *σ*_n_^eff^ the effective normal stress acting on the fault) is between 0.60 and 0.85 for most rocks (Byerlee’s rule; except for phyllosilicates-rich rocks [Byerlee 1978]), for normal stresses up to 2 GPa, and temperatures up to 780 K. The apparent friction can thus be expressed as a function of slip velocity and a state variable, and modelled with the empirical rate- and state-dependent friction law (Dieterich 1979; Ruina 1983). Additionally, at velocities typical of earthquake nucleation phase, the apparent friction varies only a few percents for small changes in slip velocity, determining if a fault is or not prone to nucleate earthquakes.

Although Byerlee’s rule and the rate-and-state law have many applications in earthquake mechanics (inter-seismic and nucleation phase of earthquakes), these experiments were performed at slip velocities and displacements orders of magnitude smaller than those of earthquakes. Therefore, these experiments are unable to characterize the propagation phase of earthquakes. In the last 15 years, the multiplication of the rotary shear apparatus, designed to achieve slip velocities higher than 1 m/s and infinite displacement, overcome those limitations and produced unexpected results (Di Toro et al. 2010). A pioneering rotary shear apparatus capable of achieving seismic slip velocities up to 1.3 m/s were built and installed in Japan (Shimamoto 1994). Amongst others (see Di Toro et al. 2010 and references therein), a state-of-art rotary shear apparatus (SHIVA, Slow to High-Velocity Shear Apparatus) capable of deforming samples at slip rates up to 9 m/s has been installed at INGV in Rome (Italy) (Di Toro et al. 2010). Studies performed with these rotary shear apparatuses have shown a significant decrease in fault strength with increasing slip and slip velocity. They also reveal various dynamic fault‐weakening mechanisms (frictional melting, thermal pressurization, silica gel, elastohydrodynamic lubrication) that are likely active during earthquakes, including mechanisms that were unknown before conducting these experiments.

Though this new frontier is promising, key aspects of earthquake mechanics laboratory investigation, like being able to conduct high slip velocity experiments on rocks under elevated pore fluid pressure and temperatures characteristic of natural and induced earthquakes, remain beyond current experimental capabilities. Furthermore, studying links between pore‐fluid pressure, permeability, and frictional properties remains a challenge. To date, very few high-velocity friction experiments have been performed in presence of pore fluid pressure (Tanikawa 2012a, b, 2014; Violay et al. 2014, 2015, 2019; Cornelio et al. 2019a, b).

In this paper, we present a new state-of-art apparatus combining the advantages of biaxial apparatuses, i.e., simple geometry, high normal forces, confining pressure and pore fluid pressure, and the advantages of the rotary shear apparatuses, i.e. high slip velocity implemented thanks to the presence of electromagnetic motors. Building on the design of recent low-velocity biaxial machines implemented with pressure vessels (Samuelson et al. 2009; Collettini et al. 2014) and implementing the system with powerful linear motors (Di Toro et al. 2010), the new HighSTEPS (High Strain TEmperature Pressure Speed) apparatus is able to reproduce the deformation conditions typical of the seismogenic crust, i.e., confining pressure up to 100 MPa, slip velocity from 10^–5^ to 0.25 m/s, temperature up to 120 °C, pore pressure up to 100 MPa. Under these unique boundary conditions, the new apparatus allows the investigation of the entire seismic cycle (inter-seismic, nucleation and propagation).

## Design of the Apparatus

**Fig. 1:**
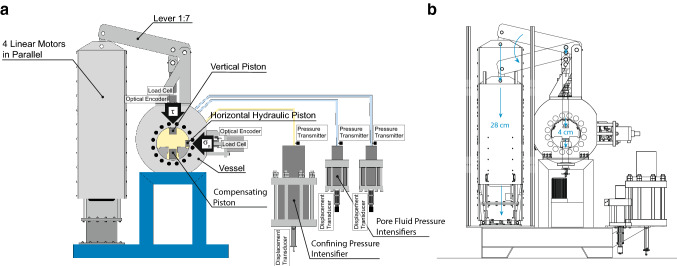
**a** Schematics of the HighSTEPS apparatus. 4 linear motors are mounted in parallel to apply vertical force to the sample through a 1:7 lever. The vertical piston is equipped with an optical linear encoder and a load cell. The horizontal hydraulic piston applies horizontal load and it is equipped with a linear optical encoder and a load cell. Two pore pressure intensifiers apply pore fluid pressure and they are equipped with displacement transducers and pressure transmitters. The confining pressure intensifier applies confining isotropic pressure and it is equipped with a displacement transducer and two-pore pressure transmitters. The intensifiers are connected to the pressure vessel. **b** Sketch of the working principle of the lever 1:7 that imposes the shear displacement and shear stress to the experimental fault

**Fig. 2:**
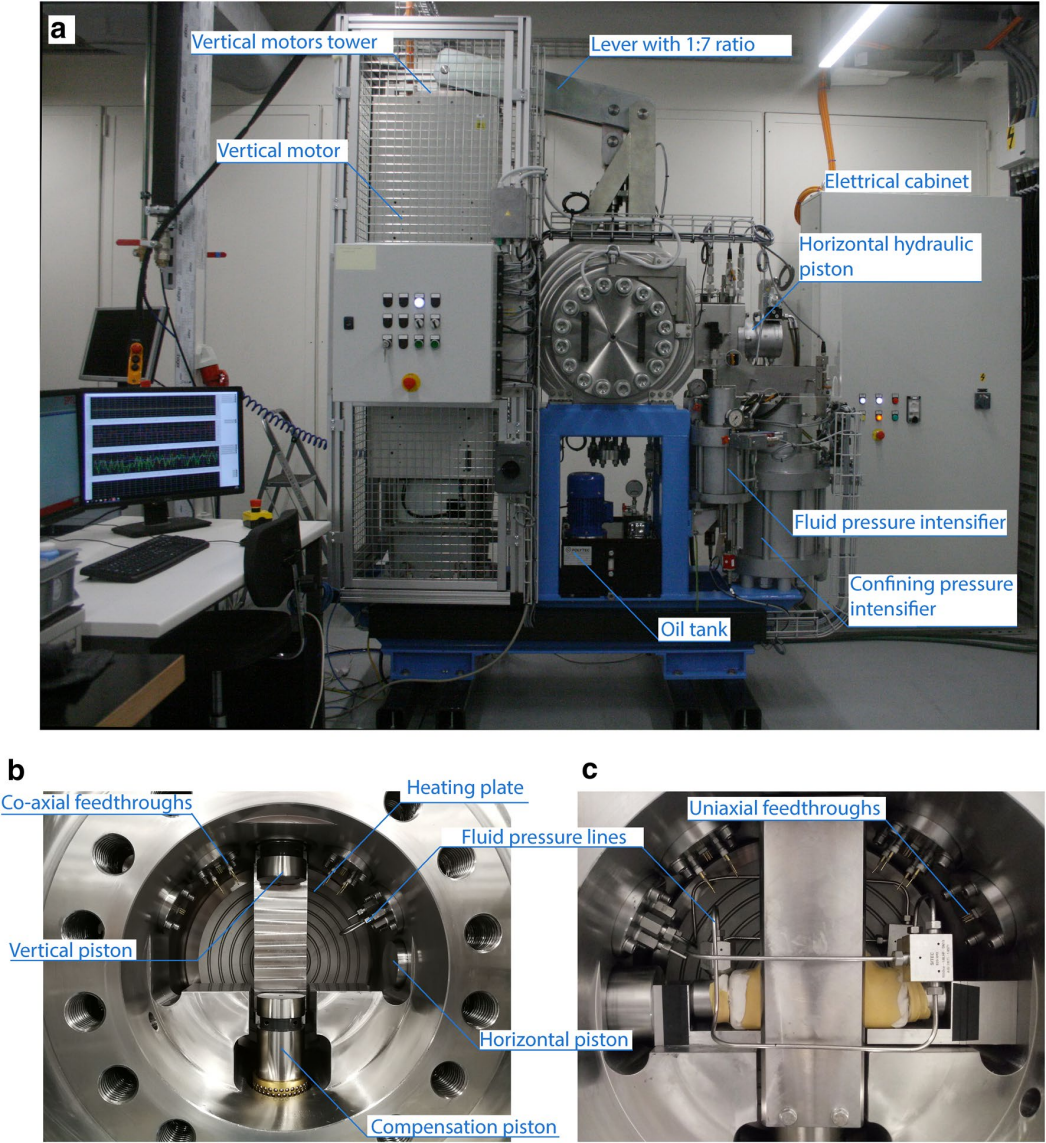
**a** Picture of the HighSTEPS machine. **b** Details of the pressure vessel with high-pressure ports for pore fluid and confining oil, and uniaxial and coaxial feed throughs for electronics. **c** Jacketed sample with pore pressure lines connected to the pore pressure ports

The machine is 1.90 m long, 0.7 m wide and 2.5 m high, and it weighs around 3000 kg (Table 1). The apparatus consists of a hydraulic system integrated with four linear motors (Figs. 1, 2a). The normal stress is applied by a horizontal hydraulic piston. The confining pressure is applied through a confining medium (i.e., silicon oil) by a hydraulic intensifier connected to a vessel implemented within the biaxial frame. The pore fluid pressure is applied by two-pore fluid intensifiers connected to the sample, which also allow for permeability measurements. In addition, the vessel is equipped with two heating plates and feed throughs for acoustic sensors and strain gauges. The main peculiarity of this apparatus is the system of four linear motors mounted in parallel to drive the vertical piston and apply to the samples shearing velocities up to 0.25 m/s, accelerations up to 10 m/s^2^ and shear stresses up to 100 MPa (Table 1).

**Table 1:** Summary of the apparatus size and maximum boundary conditions

HighSTEPS	Units	
Weight	kg	3000
Length	m	1.9
Height	m	2.5
Width	m	0.7
Max normal load	kN	160
Max shear load	kN	193
Slip rate range	m/s	10^−5^–2.5*10^−1^
Max acceleration	m/s^2^	10
Max displacement	cm	5
Max confining pressure	MPa	100
Max fluid pressure	MPa	100
Max fluid flow	cm^3^/min	60
Pore fluid Intensifier volumes	mm^3^	130
Max temperature	°C	120

### The Hydraulic Power Unit and Oil Circuit

The hydraulic power unit, with a weight of 300 kg, has a size of 1400 × 850 × 900 mm^3^ and it is located in a room next to the laboratory, about 5 m away from the machine. It was built by Polytec, S.a.s, located in Padua, Italy. It supplies pressure to the oil circuit connected to the hydraulic intensifiers and piston of the machine and includes a main oil pump driven by a 7.5 kW electrical motor, and a recycling oil pump, an oil tank, oil and air filters, a pressure accumulator to stabilize the pressure, pressure and level sensors, and finally an electro-valve allowing pressure regulation in the oil circuit. The main pump is characterized by a maximum pressure of 160 bars and a maximum flow rate of 23 l/min. The oil tank of a capacity of 75 l contains mineral oil is equipped with the recycling oil pump connected to a cooling system composed of a chiller and a heat exchanger. The recycling oil pump is characterized by a maximum pressure of 8 bars and a maximum flow rate of 24.5 l/min, respectively.

### The Three Hydraulic Intensifiers and the Hydraulic Piston

**Fig. 3:**
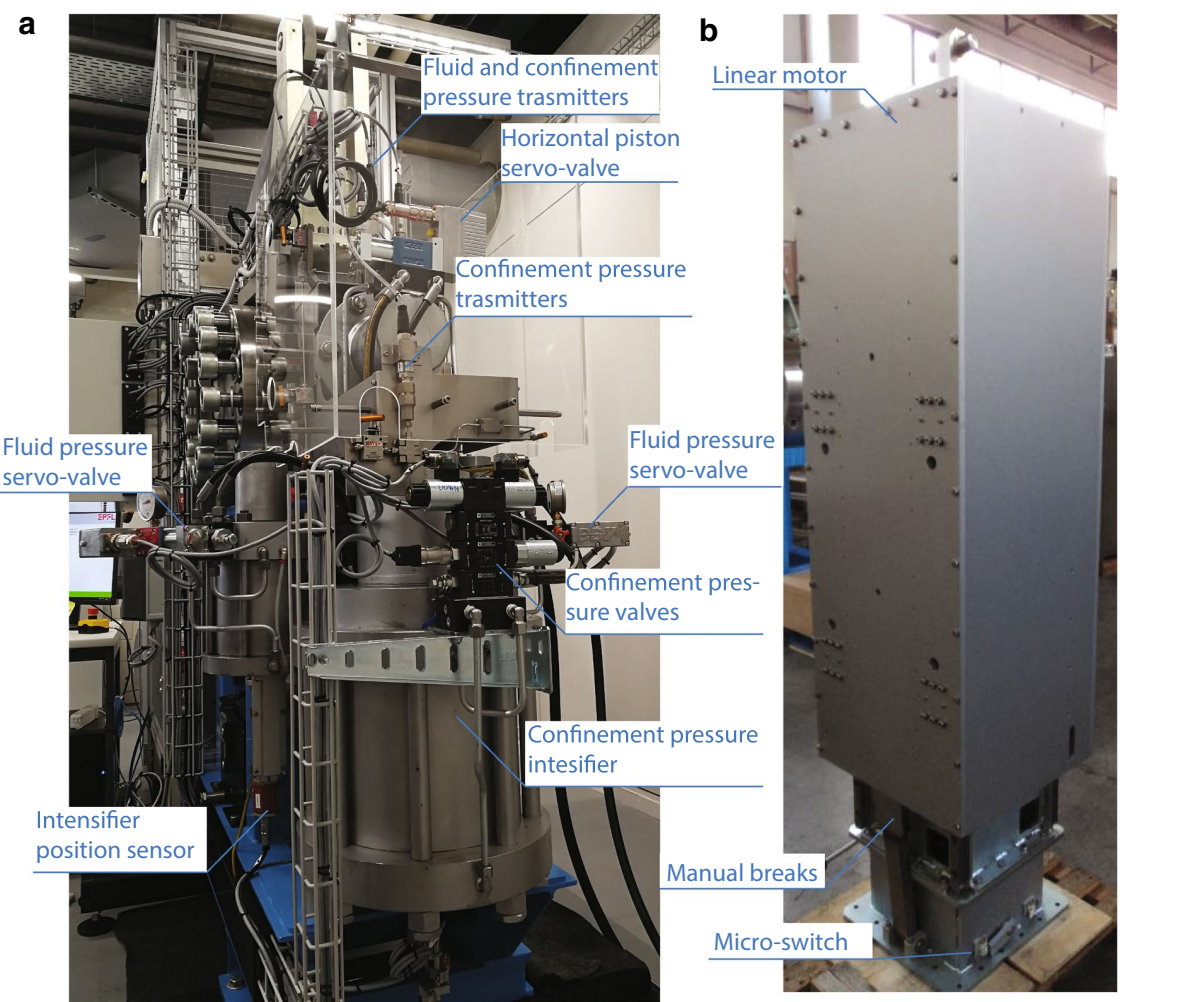
**a** Details of the intensifiers, and **b** details of the motors

The three hydraulic pressure intensifiers and the hydraulic piston were designed and built by Polytec, S.a.s., located in Padua, Italy (Fig. 3a). They work with a supplied pressure of 70–160 bar generated by the hydraulic power unit.

The horizontal hydraulic piston, with a stroke of 30 mm, is fixed on the pressure vessel and it can exert a force up to 160 KN. The piston is controlled by a servovalve (MOOG D638-390–0001 Type R02TO1M0HEE2MAOK1B1) mounted on the piston (Fig. 3a). The piston is equipped with a linear optical encoder Renishaw (RL 26BSS005C30 A) mounted on a single-track scale (RSLA ABS) which allows displacement measurement with a resolution of 5 nm. The horizontal force is measured thanks to a load cell (FUTEK LCM 550) located in the middle of the piston with a resolution ± 0.03 kN over the range 0–220 kN.

Two hydraulic intensifiers are used for fluid pressure generation up to 100 MPa, fluid flows up to 60 cm^3^/min through the sample, and dilatancy and permeability measurements. The intensifiers volume is 130 mm^3^ each. Permeability can be measured by steady state, transient and harmonic flows methods. The two intensifiers are controlled by servo-valves (MOOG D633-592B Type R01KO1D0NSS2) mounted on the top of the intensifiers. Fluid pressure is measured by a pressure transmitter (Gefran TSPA series) with a resolution of 100 kPa. The intensifiers displacement is measured by two magneto-restrictive transducers (TEMPOSONIC RP-V-0100 M-D70-1-S1B1100) with a resolution of 0.5 µm.

The third intensifier is used to apply oil confining pressure up to 100 MPa and has a larger volume than the pore fluid intensifiers, i.e., 1425.5 cm^3^. Confining pressure intensifier is controlled by a bi-directional valve (D DS3-S3/11 N-D24K1) and a single-stage proportional valve (MZE4/58–24, MVPP-D/50, MERS-GD/50) fixed on the intensifier. Confining pressure is measured by two pressure transmitters (Gefran TSPA series) with a resolution of 100 kPa, one located close to the intensifier, and the other one located close to the pressure vessel (Fig. 3). Intensifier displacement is measured by Gefran ICC150EM linear potentiometer with a stroke of 150 mm.

### Pressure Vessel

**Fig. 4:**
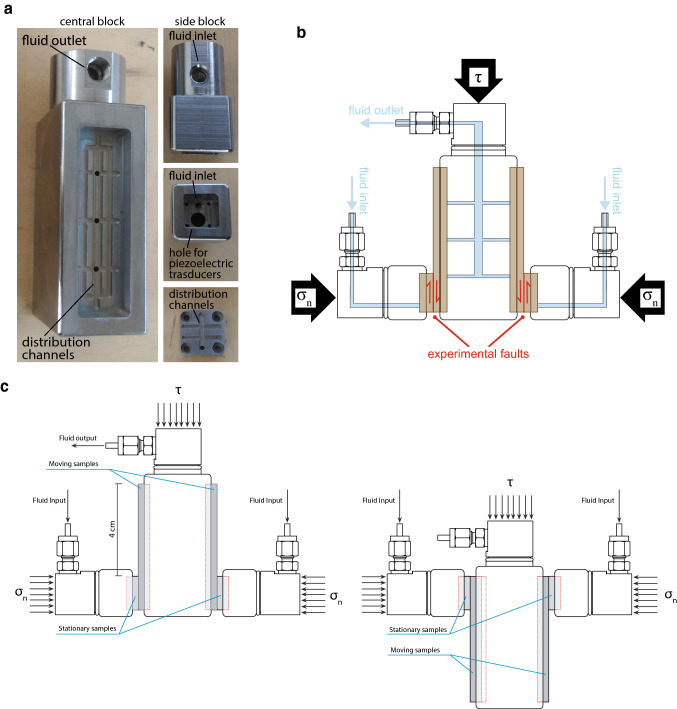
Experimental double direct shear configuration: **a** pictures of the central block showing the fluid inlet and the distribution channels, pictures of the side block showing fluid inlet, hole for piezoelectric transducer and distribution channels. **b** Schematics of the experimental double-direct-shear configuration. The sample holders are equipped with internal pore fluid channels for application of pore fluid pressure, injection of fluids and measurement of permeability/dilatancy of the fault rocks. **c** Sketch of the working principle of the double-direct shear configuration

The pressure vessel was built by RMP S.r.l., located in Rome, Italy. It is made in stainless steel, weighs about 500 kg, and has an external diameter of 700 mm and an internal diameter of 300 mm (Fig. 2b). It is designed to support 100 MPa confining pressure. The vessel holds up the vertical and horizontal pistons. To close the vessel, two doors of 130 kg each are equipped with 20 M36-size bolts. To ensure perfect sealing of the chamber, high-pressure and temperature dynamic seals are mounted on each door and on the pistons. To ensure easy opening and closing, the doors are supported by swing arms. Three pore pressure lines (two connected to one pore pressure intensifier and one connected to the other pore pressure intensifier, see Figs. 2b, c and 4a) and one oil confining pressure line are connected to the pressure vessel. The pressure vessel is equipped with eight high-pressure co-axial feed throughs from Kemlon for acoustic sensors connection, 24 uniaxial feed throughs for strain gauge connection, and 3 type K thermocouple feed throughs (Fig. 2b and c). Another access port located at the bottom of the vessel is used to fill and empty the vessel with the confining oil. The confining medium is a silicon oil from Green Star High Tech lubricants. The oil tank is equipped with a pump which is used to fill the pressure vessel above (Fig. 2a).

### Heating System

The heating system, composed of two heating plates of 26 mm diameters with a high resistance, are fixed on the inner part of the vessel doors (Fig. 2b and c). The maximum temperature of 120 °C is limited by the confining oil flash point and the maximum temperature supported by the seals located on the vessel doors. Temperature is measured within the 2 heating plates and in the confining medium by 3 K-type thermocouples. Temperature is controlled by a closed-loop regulation.

### The Four Linear Motors and Vertical Piston

The motion of the vertical piston of the machine (applying the shearing velocity and shear force to the experimental fault) represents the most innovative aspect of the apparatus. It consists in four linear motors from Kollmorgen, Type IC44-200 A3 AC TS C1, which are mechanically mounted and electrically connected in parallel (Fig. 3b). These motors are controlled by four motor controllers (one master and 3 slaves), Brushless Drive KOLLMORGEN type AKD 48A-AKD-10038. Each motor is equipped with 1 optical linear encoder Renishaw (RL 26BAT050B30A) with a resolution of 50 nm mounted on a single track-scale RSLA ABS, for a total of 4 encoders (1 master and 3 slaves) used to control the displacement, velocity and acceleration of the vertical piston. The Kollmorgen motors are frameless permanent magnet, three-phase brushless servo motors composed of a coil assembly (also called the slider) and a magnet track (also called the magnet way). The mechanical support for the four motors is composed of a fixed frame where the coil assembly are screwed resulting in a total weight of 185 kg, and a moving part where 4 aluminum plates of 35 kg each are equipped with the permanent magnet tracks. A safety block composed of two manual breaks and micro-switches is fixed at the bottom of the frame and allows motor parking between the experiments. A second safety block, composed of two rigid springs, is fixed at the top of the frame and allows safe deceleration of the motors. The motors are water cooled during long term experiments to avoid overheating of the magnetic coils. To turn on the motors, the machine is equipped with its own 125 A electrical panel. The connection between the motors and this panel is made of 4 electrical power cables and Ethernet cables for the four optical encoders (one for each motor). The four motors allow a nominal force up to 28 kN (6.9 kN each motor) and a peak force up to 37 kN (8.4 kN each motor), velocities from ~ 7 µm/s to 2.9 m/s and acceleration up to 70 m/s^2^. The motors are able to imposed a velocity function with a given acceleration, deceleration and target velocity.

The vertical piston driven by the linear motors is fixed on the pressure vessel and connected to the motors frame with a lever of 1:7 ratio (Figs. 1 and 2a), allowing a maximum force applied on the sample of 193 kN with a maximum stroke of 5 cm. However, due to the lever, velocity and acceleration on the sample side are reduced to maximum of 0.25 m/s and 10 m/s^2^, respectively. The vertical piston is equipped with an optical linear encoder Renishaw (RL 26BSS005C30 A) mounted on a single track-scale RSLA ABS which allows displacement measurement with a resolution of 5 nm. The vertical force is measured and controlled thanks to a load cell (FUTEK LCM 550) mounted in series with the piston with a resolution ± 0.03 kN over the range 0–220 KN. The vertical piston located in the upper part of the vessel is equipped with a compensation piston (co-axial and passive) in the lower part of the vessel, to avoid confining oil overpressure during fast vertical movements. The compensation piston is mechanically connected to the vertical piston thanks to 2 metallic clamps inside the pressure vessel (Fig. 2b, c). During shearing experiments, the vertical piston moves downward entering in the vessel and contemporaneously the compensation piston moves downward exiting the vessel, resulting in oil volume and oil pressure kept constant inside the vessel during the entire experiment. Moreover, the mechanical connection between the vertical piston and the compensation piston during the entire experiments ensures that the confining pressure does not contribute to the vertical load measured by the load cell. The sample assembly is located between the vertical and the compensation pistons (Fig. 2b).

### Sample Holders

Experiments can be carried out on both bare surface’s samples and powdered samples, for which two different sample holders are used. The sample holders are designed for double-direct shear configuration and are composed of three forcing blocks of stainless steel: a central block of dimension 110 × 50 × 34 mm^3^ and 2 side blocks of dimension 69 × 34 × 34 mm^3^ (Fig. 4). A constant contact area of 34 × 20 mm^2^ for bare surfaces and 34 × 34 mm^2^ for rock powder is kept constant during experiments. For experiments with powdered samples, the forcing blocks are grooved allowing shearing within the sample and not at the boundary between the sample and the forcing blocks. For bare surfaces, the forcing blocks present housing of the exact size of the samples to keep well-aligned during shearing. The forcing blocks are equipped with high-pressure fluid ports and channels allowing high pore fluid pressure experiment (Fig. 4a), and permeability and dilatancy measurement during shearing. The two side blocks are also equipped with holes for piezoelectrical transducer of 9 mm diameters. For experiments performed with confining pressure, the samples are isolated from the confining medium by using a double layer of latex jackets. This jacketing ensures limited biasing in terms of friction and can handle a large amount of deformation (maximum of 3 cm slip) before the jacket failure.

### Control and Acquisition Systems

The control and acquisition system was built by MEquadrat, based in Root, near Lucerne, Switzerland. It consists of a real-time IO Controller CompactRIO (National Instruments), which allows data acquisition at rates up to 50 kHz and real-time control of the normal stress, confining pressure, pore fluid pressure, temperature and slip velocity or shear stress. Additionally, up to 4 quarter-bridge strain gauges can be measured.

The horizontal piston can be controlled both in position mode and in force mode thanks to closed-loop servo control. The two-pore fluid pressure intensifiers can be controlled in position, flow and pressure mode thanks to the closed-loop servo control. Additionally, it is possible to impose sinusoidal oscillations of pressure. The confining oil pressure intensifier can only be controlled in pressure feedback servo control mode.

The vertical piston is controlled by a dedicated motion controller, which is controlled by the real-time IO Controller, allowing very short regulation times. The piston can be controlled in position, velocity and force mode thanks to closed-loop servo control.

## First Test of the Machine

### Stiffness Calibration

**Fig. 5:**
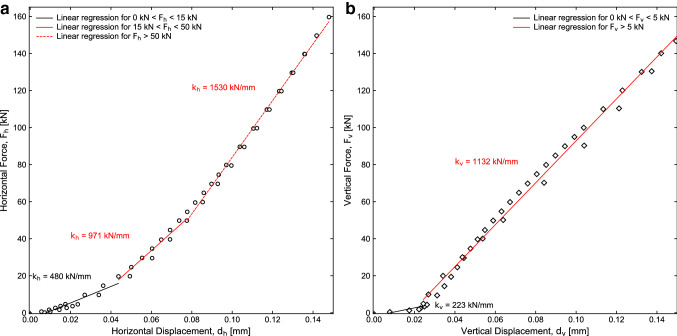
**a** Horizontal and **b** vertical machine stiffness measure with steel blocks. The horizontal stiffness is 1530 kN/mm for vertical force (*F*_h_) higher than 50 kN. The vertical stiffness is 1132 kN/mm for horizontal force (*F*_v_) higher than 5 kN

To determine the distortion of the piston during deformation of samples, we deformed steel blocks of known stiffness (Young modulus *E* = 210 GPa) with both the vertical and horizontal pistons. We measured the resulting displacement of the apparatus by removing the contribution of the elastic deformation of the steel blocks (Fig. 5). This allows us to remove from the displacements measured by the optical encoders (Fig. 1) the contribution of the pistons and evaluate fault displacement and dilation/compaction. We performed the tests under room pressure and temperature conditions, imposing force steps of 1 kN at forces below 5 kN, steps of 5 kN at forces below 80 and 100 kN, for the horizontal and vertical piston respectively, and steps of 10 kN at higher forces. After reaching 160 kN, we performed down-steps in force. Figure 5 shows the displacement versus load for both up-steps and down-steps measurements. Machine stiffness is defined as the slope of the linear regression of the points. Horizontal piston stiffness is 480 kN/mm at load < 15 kN, 971 kN/mm at load between 15 and 50 kN, and 1530 kN/mm at load > 50 kN. Vertical machine stiffness is 223 kN/mm at load < 5 kN, and 1132 kN/mm at load > 5 kN. Low stiffness at low normal stresses could be due to bad closure of the interfaces between the steel block and the piston/vessel.

### Frictionless Surfaces Calibration

**Fig. 6:**
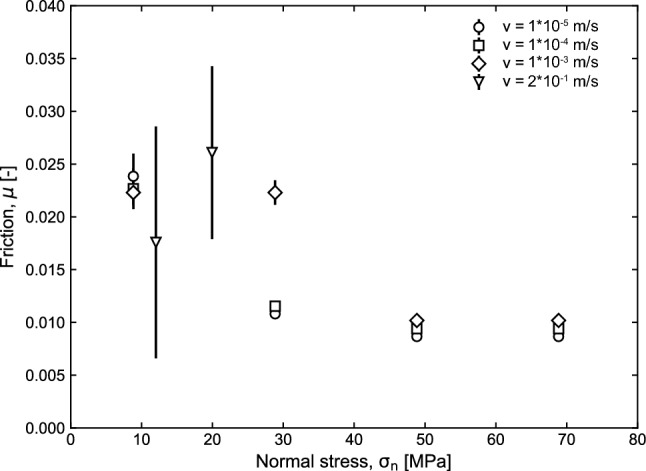
Calibration of the frictionless surfaces (GLYCODUR®) that contribute to friction measurements during experiments. The contribution of the geometrical configuration in friction is low, *µ* < 0.025

To determine the contribution of the apparatus to friction measurement on rock samples, we performed frictional tests with frictionless surfaces consisting of GLYCODUR®, that is PTFE-based 3-layer material. To evaluate the machine contribution to friction two GLYCODUR plates were mounted in double-direct sample holders. We measured the resulting shear stress versus displacement evolution. Tests were performed at room pressure and temperature conditions, imposing normal stress between 10 and 70 MPa, and sliding velocities between 10^−5^ and 2*10^–2^ m/s. Figure 6 shows the resulting friction versus normal stress at different velocities. Machine contribution to friction is in general extremely low with a maximum value of µ = 0.020–0.025 at low normal stress and high velocity. We can use GLYCODUR surfaces when we shear rock bare surfaces in single-direct shear configuration.

### Preliminary Results on Calcite and Quartz

#### Effect of Normal Stress

We used both gouge and bare surfaces of calcite (Carrara marble) to test the normal stress dependence of the frictional strength and compare the measurements performed with the new machine with previous experiments. Experiments were run under double-direct shear configuration for gouge and single direct shear for bare surfaces.

To produce calcite gouge, Carrara marble was crushed and sieved to < 125 µm grain size. Two gouge layers of 4 mm thickness were sandwiched between the grooved steel blocks (see paragraph II F). To produce calcite bare surfaces samples, Carrara marble slabs of 12 mm thickness were cut into pieces with a dimension of 20 × 34 × 12 mm^3^ and 70 × 34 × 12 mm^3^, to ensure a 34 × 20 mm^2^ constant contact area during shearing. The two surfaces in contact during the experiment were polished with a P80-grit diamond abrasive disc. Experiments were performed without confining pressure, under room temperature and room humidity conditions.

Steady-state friction coefficient was determined at a slip velocity of 1*10^–5^ m/s and normal stresses from 10 to 30 MPa, for both gouge and bare surfaces.

**Fig. 7:**
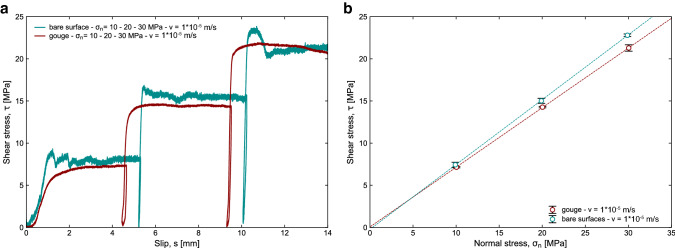
Calcite (Carrara marble) gouge and bare surfaces frictional experiments: **a** shear stress versus slip during frictional sliding at *v* = 1*10^–5^ m/s and normal stresses from 10 to 30 MPa. Experiments were performed under double-direct shear configuration on gouge and single-direct shear on bare surfaces. **d** Steady-state shear strength versus normal stress for both gouge material and bare surfaces. Data plot along a single line with a slope of 0.77 for bare surfaces and 0.71 for gouge, in agreement with Byrelee's rule (Byerlee 1978)

The shear stress versus displacement curves are shown Fig. 7a. In all experiments, the shear stress first increased linearly with the displacement (i.e., elastic phase). After the elastic phase, gouge samples show a nonlinear decreasing of shear stress as a function of displacement (i.e., slip hardening phase) until a constant shear stress (i.e., steady-state phase) is achieved steady-state. Bare surface samples show a shorter slip hardening phase followed by a peak shear stress and a steady-state shear stress. Figure 7b shows the linear pressure dependence of calcite frictional strength (e.g., Byerlee 1978). The steady-state friction coefficient is obtained from the linear regression of the steady-state shear stress versus applied normal stress and it is 0.77 for bare surfaces and 0.71 for gouge. The intercept of the linear regression shows the negligible cohesion of the bare surfaces (− 0.22 MPa) and the gouge (0.07 MPa). The steady-state apparent friction during experiments at low velocity (1*10^–5^ m/s) and 10 MPa normal stress is µ ≈ 0.7 in agreement with previous studies (Verberne et al. 2014; Carpenter et al. 2015, 2016; Chen et al. 2015; Acosta et al. 2020).

#### Effect of Velocity

**Fig. 8:**
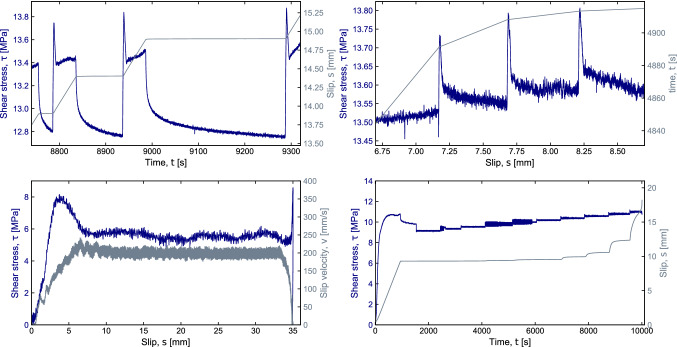
Example of velocity control and shear stress control experiments performed: **a** slide-hold-slide test performed on calcite gouge at 10 MPa normal stress under room temperature and room humidity conditions; the hold periods last 30, 100, and 300 s; **b** velocity steps (1*10^–5^–3*10^–5^–1*10^–4^–3*10^–4^ m/s) on calcite gouge at 20 MPa normal stress under room temperature and room humidity conditions; **c** high slip velocity friction test on gabbro bare surface at 10 MPa normal stress under room temperature and room humidity conditions; **d** shear stress control test on calcite gouge at 20 MPa normal stress under room temperature and room humidity conditions

Faults respond to perturbations depending on their stability state by remaining locked (stage 1), undergoing slow and stable sliding (stage 2), experiencing local on-fault short-lived unstable events (earthquake precursory sequence) (stage 3), or accelerating toward catastrophic seismic slip (main event) (Kaneko et al. 2010) (stage 4). The transition from stage 1 to 2, 3 or 4 controls the slip behavior during fault reactivation, i.e., the earthquake potential of a fault. Rate- and- state friction (RSF) laws provide a comprehensive analysis of the slip behavior. In this framework, the frictional response of a fault varies with the previous loading history and depends on both the instantaneous slip velocity, normal stress, and a state variable that describes the progressive evolution of the sliding interfaces (Dieterich 1972; Ruina 1983; Dieterich and Linker 1992). Depending on the frictional evolution in response to an instantaneous change in slip velocity or normal stress, the rate-and-state law evaluates the capability of a fault to nucleate earthquakes or to creep aseismically. Stage 4 controls earthquake propagation. During this stage, the slip velocity and power density (shear stress by velocity) increase drastically, inducing strong fault frictional weakening. Frictional weakening is controlled by a number of processes, such as flash heating and melting (Rice 2006), decomposition reactions (Han et al. 2007), and superplastic flow and thermal pressurization (Violay et al. 2015). Many of these processes are actually thermally triggered (Di Toro et al. 2011 and references therein). Currently, (1) how the transition from slow slip velocity (RSF, slip velocity ~ μm/s) to high-velocity weakening behavior (slip velocity > cm/s) occurs, and (2) the conditions that drive faults through the aforementioned stages 1 to 4 are not clear. The HighSTEPS apparatus coverslip velocity from μm/s to m/s (i.e., stages 1 to 4), enabling the measurement of the rate- and- state friction parameters and friction evolution during fault weakening and lubrication. Therefore, a complete collection of the mechanical data that are required to assess a constitutive equation for rock-friction will be possible. In Fig. 8, examples of a slide-hold-slide sequence (Fig. 8a), a velocity step sequence (Fig. 8b), high-velocity friction experiment (Fig. 8c) and shear stress control experiment (Fig. 8d) are shown.

Slide-hold-slide sequences are performed to measure friction healing after a period of hold and thus to simulate fault re-strengthening during the inter-seismic phase. During this sequence, calcite gouge layers were sheared at a constant velocity of 10^–5^ m/s followed by a hold period (t_h_) during which the vertical ram stopped and gouge layers were under quasi-stationary contact. The slide-hold-slide sequence was conducted under room temperature and room humidity conditions. The hold periods showed in Fig. 8a are 30, 100 and 300 s. After each hold period, the gouge was re-sheared at 10^–5^ m/s. As is shown in Fig. 8a. We observe an increase of friction upon re-shear, followed by a decay to the previous steady-state value. This difference in friction between the peak and the steady state (Δµ) is defined as the frictional healing and is typical of granular gouge material (e.g., Marone 1998; Richardson and Marone 1999). The frictional healing rate β = Δµ/Δlog_10_(*t*_h_) measured for calcite gouge under dry conditions is *β* = 0.004 in agreement with previous works [e.g., Chen et al. 2015].

The velocity steps (1*10^–5^–3*10^–5^–1*10^–4^–3*10^–4^ m/s) were performed on calcite gouge with grain size < 125 µm under room temperature and room humidity conditions (Fig. 8b) and showed an abrupt change in friction (direct effect, *a* parameter in rate-and-state law) and a pronounced evolution effect (*b* parameter in rate-and-state law). The resulting *a–b* values, i.e., slightly velocity strengthening/velocity-neutral behavior, are in agreement with previous studies on calcite gouge under dry conditions (e.g., Chen et al. 2015).

High-velocity friction experiment was performed on gabbro bare surfaces (initial roughness applied with a P80-grit diamond abrasive disc) at slip rate of 0.2 m/s, acceleration and deceleration of 5 m/s^2^ and normal stress of 10 MPa (Fig. 8c). Once the velocity function was applied, the sample initially deformed elastically (i.e., the shear stress increased linearly with time), until the static friction was overcome and slip on the sample initiated. Consistently with previous experimental observations, the shear stress decayed from a peak value (*µ* = 0.9) towards a steady-state shear stress value of 6 MPa corresponding a steady-state friction coefficient of 0.6 (e.g., Tsutsumi and Shimamoto 1997).

The vast majority of previously described experiments, either under slow or high slip velocity, has been conducted by imposing velocity functions. However, it is more realistic boundary condition to describe fault loading in terms of acting stress, whether it is virtually constant, slowly increasing due to tectonic loading, or increasing/decreasing in sudden steps (stress transfer) due to ruptures in the vicinity of the fault. Thus, controlling the shear stress and allowing the slip velocity to adjust spontaneously, rather than the contrary, is closer to natural conditions where the “far field” stress, together with the frictional properties of the fault materials, controls the mechanical response of the fault zone. To this end, HighSTEPS apparatus is able to impose up and down shear stress steps. Figure 8d shows an experiment conducted in shear stress control mode. Initially, the calcite gouge sample was deformed under double-direct shear configuration at a constant velocity of 10^−5^ m/s and normal stress of 20 MPa, room pressure and temperature condition, until the steady-state shear stress was achieved and the fault accumulated 10 mm of displacement. Then we switched the controlled mode into shear stress control mode, and the shear stress was gradually increased by small (0.5 MPa) stepwise increments. The response to the loading is measured in terms of slip velocities. After each shear increment, we waited until either a quasi-static balance or a steady-state sliding is achieved before applying of the next stress increment. The process is repeated until the onset of the main instability, that is, the catastrophic acceleration of slip to 10^–4^ m/s. We observe slip pulses which develop right after the instantaneous shear stress increase. During the last stress step, the fault gouge spontaneously evolved from primary to secondary, and tertiary creep (Kassner and Pérez-Prado 2004).

#### Test of the Confining Pressure, Pore Fluid Pressure and Temperature

**Fig. 9:**
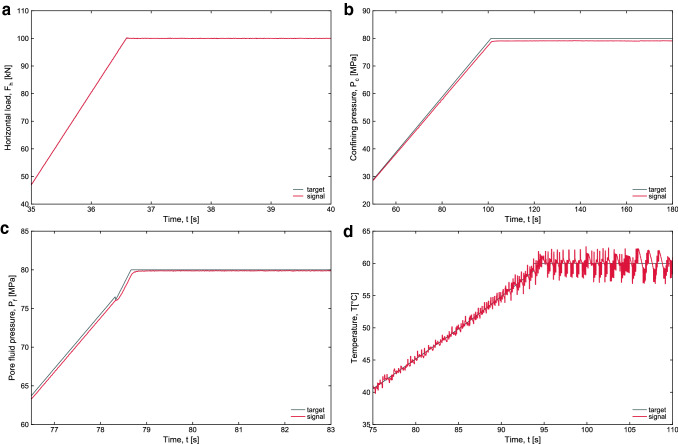
**a** Horizontal load versus time showing the load control in ramp (kN/s) and constant value (100 kN). **b** Confining pressure versus time showing the control in pressure up to 80 MPa. **c** Pore fluid pressure versus time showing the control in pore fluid pressure. **d** Temperature versus time showing the control in temperature up to 60 °C

First, tests conducted within the pressure vessel, applying confining pressure, pore pressure and temperature have been performed without shearing the sample, i.e., without vertical motion. Figure 9 show that the vessel and the confining and pore fluid intensifiers can support pressure up to 80 MPa testifying the accurate control of these parameters. Figure 8d shows also that temperature control is accurate (± 3–4 °C).

In a second time, we conducted a test where we sheared bare surfaces of gabbro (initial roughness applied with a P80-grit diamond abrasive disc) in single-direct shear configuration using the forcing blocks (Fig. 2c), the frictionless surface (Paragraph III B) and the jacketing system. The constant experimental conditions were: *σ*'*n* = 50 MPa, confining pressure, Pc = 30 MPa, pore pressure of 5 MPa, temperature 25 °C, sliding velocity 1*10^–2^ m/s, and total displacement 1*10^–2^ m.

The procedure for experiments performed in the presence of confining g pressure and pore fluid consisted of the following steps: (1) we applied the 10 MPa of normal stress; (2) we filled the vessel; (3) we increased stepwise the applied normal stress and the confining pressure to *σ*_*n*_ = 50 MPa and *P*_c_ = 10 MPa; (4) we saturated the sample and we increased the pore fluid pressure to P_f_ = 5 MPa; (5) we increased the stepwise the normal stress and the confining pressure to the target values, i.e. *σ*_*n*_ = 55 MPa and *P*_c_ = 30 MPa; (6) we isolated the pore pressure intensifiers from the sample (undrained condition); (7) we applied the shear stress advancing the vertical piston at a constant velocity of 1*10^–2^ m/s. Since the area of the forcing piston is larger than the sample contact area (A) and the load cell is located outside the vessel, the confining pressure contributes to the applied horizontal force (*F*_h_) measured by the horizontal load cell and the effective normal stress is evaluated as follow:

1 $${\sigma }_{n}=\frac{{F}_{h}-{2.023P}_{c}}{A}-{P}_{f}$$

**Fig. 10:**
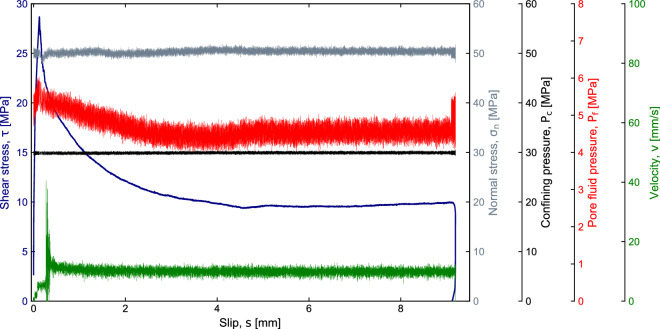
Shear experiments conducted on gabbro bare surface under unique experimental conditions: confining pressure of 30 MPa, effective normal stress of 50 MPa, undrained pore fluid pressure of 5 MPa and slip velocity of 1*10^–2^ m/s

In Fig. 10, we show the evolution of the shear stress, effective normal stress, confining pressure, pore fluid pressure and slip velocity with displacement during the experiment. We observe an abrupt initial strengthening until a peak friction (*µ* = 0.6) followed by a strong weakening towards a steady-state value around 0.2. This experimental observation is in accordance with previous high-velocity friction tests on gabbro (Tsutsumi and Shimamoto 1997; Violay et al. 2019). The extremely rapid drop in friction corresponds to a slip weakening distance *D*_w_ (or the distance over which a significant decrease in shear stress occurs) of about 4–5 mm. This value is smaller than the value observed at lower normal stress and without confining pressure in rotary shear machines, in agreement with a flash heating theoretical prediction (Goldsby and Tullis 2011). Experiments performed with HighSTEPS indicate that faults are lubricated after ~ 125 µm of displacement under effective normal stresses 50 and P_c_ of 30 MPa). Although what is observed in a rotary shear machine (Violay et al. 2019), we do not observe healing in apparent friction during the deceleration phase of the experiments.

## Future Machine Development

We are currently finalizing the heating system to perform experiments under high-temperature conditions and an additional jacketing system that will also allow to use up to 8 acoustic sensors glued directly on the rock samples and up to 4 quarter-bridge strain gauges. With this new equipment, we aim at better understanding the evolution of frictional properties of fault rocks over the entire seismic cycle, going from the long inter-seismic period, earthquake nucleation to propagation.

## References

[CR1] Acosta M, Maye R, Violay M (2020) Hydraulic transport through calcite bearing faults with customized roughness: effects of normal and shear loading. J Geophys Res Solid Earth 125(8): e2020JB019767

[CR2] Brace WF, Byerlee JD (1966). Stick-slip as a mechanism for earthquakes. Science.

[CR3] Byerlee JD (1967). Frictional characteristics of granite under high confining pressure. J Geophys Res.

[CR4] Byerlee, J (1978) "Friction of rocks." Rock friction and earthquake prediction. Birkhäuser, Basel, pp 615-626

[CR5] Carpenter BM (2015). Influence of calcite decarbonation on the frictional behavior of carbonate-bearing gouge: implications for the instability of volcanic flanks and fault slip. Tectonophysics.

[CR6] Carpenter BM (2016). The influence of normal stress and sliding velocity on the frictional behaviour of calcite at room temperature: insights from laboratory experiments and microstructural observations. Geophys J Int.

[CR7] Chen J (2015). Interseismic re-strengthening and stabilization of carbonate faults by “non-Dieterich” healing under hydrothermal conditions. Earth Planet Sci Lett.

[CR8] Collettini C, et al. (2014) A novel and versatile apparatus for brittle rock deformation. Int J Rock Mech Min Sci 66: 114–123.

[CR9] Cornelio C, et al. (2019a) Effect of fluid viscosity on fault reactivation and co‐seismic weakening. J Geophys Res Solid Earth

[CR10] Cornelio C, et al. (2019b) Mechanical behaviour of fluid-lubricated faults. Nat Commun 10.1: 1–7. 10.1038/s41467-019-09293-9 PMC6426875 30894547

[CR11] Di Toro G, et al. (2010) From field geology to earthquake simulation: a new state-of-the-art tool to investigate rock friction during the seismic cycle (SHIVA). Rendiconti Lincei 21.1: 95–114.

[CR12] Di Toro, G, et al. (2011) Fault lubrication during earthquakes. Nature 471.7339: 494–498. 10.1038/nature09838 21430777

[CR13] Dieterich JH (1972). Time-dependent friction in rocks. J Geophys Res.

[CR14] Dieterich JH (1979). Modeling of rock friction: 1. Experimental results and constitutive equations. J Geophys Res Solid Earth.

[CR15] Dieterich JH, Linker MF (1992) Fault stability under conditions of variable normal stress. Geophys Res Lett 19.16: 1691–1694.

[CR17] Giorgetti C, et al. (2015) Frictional behavior of talc‐calcite mixtures. J Geophys Res Solid Earth 120.9: 6614–6633.

[CR18] Goldsby DL, Tullis TE (2011). Flash heating leads to low frictional strength of crustal rocks at earthquake slip rates. Science.

[CR19] Han R, et al. (2007) Ultralow friction of carbonate faults caused by thermal decomposition. Science 316.5826: 878–881. 10.1126/science.1139763 17495168

[CR20] Handin J (1969). On the Coulomb-Mohr failure criterion. J Geophys Res.

[CR21] Jaeger JC (1959). The frictional properties of joints in rock. Geofisica Pura e Applicata.

[CR22] Kanamori H, Anderson DL (1975). Theoretical basis of some empirical relations in seismology. Bull Seismol Soc Am.

[CR23] Kaneko, Y, et al. (2010) Towards inferring earthquake patterns from geodetic observations of interseismic coupling. Nat Geosci 3.5: 363–369.

[CR24] Kassner ME, Pérez-Prado MT (2004) Fundamentals of creep in metals and alloys. Elsevier Science.

[CR25] Lee WHK, et al. (2002) International handbook of earthquake & engineering seismology. Elsevier

[CR26] Lockner, DA, Nicholas MB (2002) Rock failure and earthquakes. Int Geophys Ser 81.A: 505–538.

[CR27] Mair K, Kevin MF, Chris M (2002) Influence of grain characteristics on the friction of granular shear zones. J Geophys Res Solid Earth 107.B10: ECV-4.

[CR28] Marone C (1998). Laboratory-derived friction laws and their application to seismic faulting. Annu Rev Earth Planet Sci.

[CR30] Maxwell SC, et al. (2010) Petroleum reservoir characterization using downhole microseismic monitoring. Geophysics 75.5: 75A129–75A137.

[CR32] Paterson MS, Teng-fong W (2005) Experimental rock deformation-the brittle field. Springer Science & Business Media

[CR33] Persson, BNJ (2013) Sliding friction: physical principles and applications. Springer Science & Business Media

[CR34] Rice JR (2006) Heating and weakening of faults during earthquake slip. J Geophys Res Solid Earth 111.B5

[CR35] Richardson E, Marone C (1999). Effects of normal stress vibrations on frictional healing. J Geophys Res Solid Earth.

[CR36] Ruina A (1983). Slip instability and state variable friction laws. J Geophys Res Solid Earth.

[CR37] Samuelson J, Derek E, Chris M (2009) Shear‐induced dilatancy of fluid‐saturated faults: Experiment and theory. J Geophys Res Solid Earth 114.B12

[CR38] Savage HM, Chris M (2007) Effects of shear velocity oscillations on stick‐slip behavior in laboratory experiments. J Geophys Res Solid Earth 112.B2

[CR39] Scholz CH (1998). Earthquakes and friction laws. Nature.

[CR40] Scholz CH (2019) The mechanics of earthquakes and faulting. Cambridge University Press 10.1126/science.250.4988.1758-a 17734719

[CR41] Shearer PM (2019) Introduction to seismology. Cambridge university Press

[CR42] Shimamoto T (1994). A new rotary-shear high-speed frictional testing machine: its basic design and scope of research. J Tectonic Res Group Jpn.

[CR43] Tanikawa W, Mukoyoshi H, Tadai O (2012a) Experimental investigation of the influence 641 of slip velocity and temperature on permeability during and after high-velocity fault slip. J Struct Geol 38: 90–101. 10.1016/j.jsg.2011.08.013

[CR44] Tanikawa W, Mukoyoshi H, Tadai O, Hirose T, Tsutsumi A, Lin W (2012b) Velocity 644 dependence of shear-induced permeability associated with frictional behavior in fault zones of 645 the Nankai subduction zone. J Geophys Res Solid Earth 117(B5): B05405.646 10.1029/2011JB008956

[CR45] Tanikawa W, Tadai O, Mukoyoshi H (2014) Permeability changes in simulated granite faults during and after frictional sliding. Geofluids 14(4) 481–494. 10.1111/gfl.1209

[CR46] Tsutsumi A, Shimamoto T (1997). High-velocity frictional properties of gabbro. Geophys Res Lett.

[CR47] Verberne BA (2014). Frictional properties and microstructure of calcite-rich fault gouges sheared at sub-seismic sliding velocities. Pure Appl Geophys.

[CR48] Violay M, et al. (2014) Effect of water on the frictional behavior of cohesive rocks during earthquakes. Geology 42.1: 27–30.

[CR49] Violay M, et al. (2015) Thermo-mechanical pressurization of experimental faults in cohesive rocks during seismic slip. Earth Planet Sci Lett 429: 1–10.

[CR50] Violay M, et al. Effect of water and rock composition on re-strengthening of cohesive faults during the deceleration phase of seismic slip pulses. Earth Planet Sci Lett 522: 55–64.

